# Knock-In Mice with Myo3a Y137C Mutation Displayed Progressive Hearing Loss and Hair Cell Degeneration in the Inner Ear

**DOI:** 10.1155/2018/4372913

**Published:** 2018-07-05

**Authors:** Peipei Li, Zongzhuang Wen, Guangkai Zhang, Aizhen Zhang, Xiaolong Fu, Jiangang Gao

**Affiliations:** School of Life Science, Shandong University, Jinan 250100, China

## Abstract

Myo3a is expressed in cochlear hair cells and retinal cells and is responsible for human recessive hereditary nonsyndromic deafness (DFNB30). To investigate the mechanism of DFNB30-type deafness, we established a mouse model of Myo3a kinase domain Y137C mutation by using CRISPR/Cas9 system. No difference in hearing between 2-month-old Myo3a mutant mice and wild-type mice was observed. The hearing threshold of the ≥6-month-old mutant mice was significantly elevated compared with that of the wild-type mice. We observed degeneration in the inner ear hair cells of 6-month-old Myo3a mutant mice, and the degeneration became more severe at the age of 12 months. We also found structural abnormality in the cochlear hair cell stereocilia. Our results showed that Myo3a is essential for normal hearing by maintaining the intact structure of hair cell stereocilia, and the kinase domain plays a critical role in the normal functions of Myo3a. This mouse line is an excellent model for studying DFNB30-type deafness in humans.

## 1. Introduction

Deafness presents the highest incidence among sensory defects. One-third of the global population suffer from hearing impairment. Approximately 300 million people currently possess hearing disabilities [1–3]. Presbyacusis, also known as age-related hearing loss (ARHL), refers to the gradual onset of sensorineural hearing loss with age. According to WHO statistics, over one-third of the >65-year-old population are currently affected by senile deafness. The contribution of hearing loss to the general health of individuals during that extended lifespan is of considerable clinical and economic significance [4]. ARHL inevitably causes communication difficulties and is associated with social isolation and depression and decreases physical and cognitive function [5, 6]. Therefore, determining ways to prevent presbyacusis and identifying its pathogenesis have become crucial. Senile deafness is related to environmental factors, such as disease, ear-toxic drugs, noise, and mental trauma, and genetic factors play an important role in this process. Nearly 50% of age-related deafness is determined by genetic factors [7]. Senile deafness is a complex disease that can be affected by a variety of environmental factors, and differences in the genetic background of different populations can interfere with research. Therefore, the use of gene knock-out/knock-in mouse model can strictly control environmental conditions and reduce the influence of genetic background, thereby providing important tools for the validation of age- and deafness-related genes. Therefore, the establishment of presbyacusis animal model has become an important method in studying presbyacusis.

Our hearing depends on hair cells in the inner ear, which can convert the vibrations of sound into electrical signals. At the apical surface of each hair cell, three rows of stereocilium are arranged in high and low orders [8, 9]. When sound is introduced into the inner ear, sound waves cause a slight vibration of the cochlear basilar membrane. The basilar membrane vibration is transduced into an electric signal by a mechanoelectro transduction (MET) protein, which can open the ion channel at the top of the stereocilium and generate a membrane potential. The mechanical signal transduction that occurs at the top of the hair cell stereocilium may comprise a complex of proteins called MET protein complex [10, 11]. In mammals, Myo3a is mainly expressed in the retina [12] and cochlea [13, 14]. In inner ear hair cells, Myo3a is specifically expressed near the tips of the stereocilium, where the MET protein is located [13]. Myo3a protein localization implies its importance in hearing. Myo3a mutations in humans can cause nonsyndrome-type deafness (DFNB30) [14], where patients experience progressive bilateral hearing loss. Hearing loss usually begins at the second decade and becomes severe by the age of 50. In addition, Myo3a is a candidate gene for the disease Bardet-Biedl syndrome, an important human retinal disease [15].

Based on different C-terminal cargo-binding domains, myosin superfamily members are classified into conventional myosins (class II) and unconventional myosins (classes I and III–XV) [16–18]. Myo3a is a class III myosin. The Myo3a protein contains a kinase domain at the N-terminal, followed by a highly conserved motor region and three IQ motifs. Two of the IQ motifs are located at the conserved neck region, and the third motif is located at the center of the tail domain [19]. Myo3a is special because it is autophosphorylated by a kinase domain [20]. In 2010, Walsh et al. generated the Myo3a knock-out mouse model, which showed the characteristics of senile deafness [21]. The knock-out mouse model converts the 1041th codon of Myo3a into a termination codon, which is located at the 28th exon, allowing the knock-out of the protein after the 27th exon. However, the head kinase domain, motor domain, and the regulatory neck domain still remain. The mutation site of Myo3a in humans frequently appears in the head domain of the protein. Our collaborators and our team found that a Myo3a Y129C mutation within the kinase domain can cause ARHL in humans (data not shown). Mimicking this point mutation, we established knock-in mice by changing the tyrosine (Y137) to cysteine in Myo3a. A series of experiments was conducted on these mutant mice to determine the function of Myo3a in the cochlea. The mutant mice displayed progressive hearing loss and inner ear hair cell degeneration. The mouse we generated is a good model for studying DFNB30-type deafness in human and provides valuable material for the study of ARHL.

## 2. Materials and Methods

### 2.1. Ethics Statement

All animal experimental procedures were approved by the Ethics Committee of Shandong University. Animal management was performed strictly in accordance with the standards of the Animal Ethics of Shandong University.

### 2.2. Generation of Myo3a Y137C Mouse

Myo3a mutant mice were generated using the CRISPR-Cas9 genome-editing technology and maintained on the CBA/CaJ background. pX330 plasmid was obtained from Addgene (plasmid ID number 42230). The CRISPR-Cas9 genome-editing technology in mice was used as previously described [22–26]. In brief, a pair of oligonucleotides for the target sequence (5′-CACCGTGTAAAATATATGCAATTAC-3′ and 5′-AAACGTAATTGCATATATTTTACAC-3′) was annealed and ligated to pX330 that was previously digested with BbsI. The pX330 plasmid containing sgRNA and Cas9 was purified and eluted in RNase-free water. The following repair template, which contained the target mutation and two synonymous mutations, was commercially synthesized as follows: ggatttctgaagaggggagaaagaatgagcgagcctgtaatCgcCtGtattttacacgaagcactaatggtaaggctatttgaactctt.

CBA/CaJ female mice were superovulated and mated with CBA/CaJ male mice. The fertilized eggs were removed from the oviducts on the next day. pX330 plasmid (5 ng/*μ*L) mixed with 10 ng/*μ*L repair template was microinjected into the pronucleus of fertilized eggs. After injection, eggs were incubated for 10 min. The eggs were then transferred into the oviducts of pseudopregnant CD1 female mice.

Genomic DNA was extracted from the tails of newborn pups. The genomic DNA fragment around the gRNA target site was amplified by PCR using the Myo3a forward primer 5′-AGCTGTGACCTTTTTGAAGATAGC-3′ and the Myo3a reverse primer 5′-ATCAACAAACACCAAGCTGCC-3′. The total reaction volume of 40 *μ*L contains Taq DNA polymerase, PCR buffer, 2 *μ*L 10 mM forward primers, 2 *μ*L 10 mM reverse primer, and 1 *μ*L DNA fragment. Amplification was performed for 3 min at 95°C, followed by 33 cycles, with each consisting of 30 S at 95°C, 30 S at 60°C, and 30 S at 72°C, with a final extension step of 10 min at 72°C. The obtained PCR products were directly sequenced or cloned using the T/A cloning method and then sequenced to identify the mutation.

### 2.3. Measurement of Hearing Thresholds

ABR was measured to determine the hearing thresholds of mice in a sound-isolated room as previously described [27, 28]. Mice were anesthetized with 0.007 g/mL pentobarbital sodium by intraperitoneal injection (50 mg/kg body weight). Three needle electrodes were placed subcutaneously in the anesthetized mice. Ground, active, and reference electrodes were placed at the back near the tail, above the vertex between the eyes, and underneath the ear, respectively. Hearing thresholds were measured using a Tucker-Davis Technologies System (TDT, USA) workstation that runs the SigGen32 software (TDT, USA). Mice were presented with click and tone burst stimuli at frequencies of 4, 8, 16, and 32 kHz. Auditory thresholds (dB SPL) were determined by decreasing the sound intensities from 90 dB to 10 dB until the lowest sound intensity at which waveforms lose their reproducible morphology is reached. Myo3a mutant mice were compared with their wild-type littermates at each age. More than five animals were used in each experiment.

### 2.4. Paraffin Sectioning and Hematoxylin and Eosin (H&E) Staining

The cochleae from Myo3a mutant and wild-type mice were removed, fixed with 4% formaldehyde in 10 mM phosphate buffer at 4°C overnight, decalcified in 10% EDTA in 10 mM phosphate-buffered saline (PBS) at room temperature for 2 d, dehydrated with 30% to 100% ethanol series, and treated with xylene for transparency. The cochlea was embedded in paraffin, and the specimen was sectioned at 7 mm thickness using a thin semiautomatic microtome. Sections were deparaffinized using xylene and 100% to 30% ethanol series, stained with H&E, and viewed under a light microscope (Nikon YS100).

### 2.5. Whole-Mount Staining

Wild-type and Myo3a mutant mice were anesthetized with 0.007 g/mL pentobarbital sodium. The cochleae were removed from anesthetized mice, fixed in 4% formaldehyde in 10 mM PBS at 4°C overnight, and decalcified in 10% EDTA at room temperature for 2 days [24, 26]. We isolated the basilar membrane from the cochlea under the Nikon TE2000 fluorescence microscope. The basilar membrane was washed with 10 mM PBS and blocked in 5% goat serum for 30 min at 37°C. Primary antibodies were diluted in 10 mM PBS and incubated with the basilar membrane at 4°C overnight. After being washed with 10 mM PBS, the basilar membrane was incubated at 37°C for 1 h in anti-rabbit TRITC-conjugated secondary antibody diluted in PBS [29, 30]. The basilar membrane was washed again with PBS, and Alexa Fluor 488-conjugated phalloidin (2 *μ*g/mL, Sigma) was applied to the samples for 30 min and 4,6-diamidino-2-phenylindole (DAPI) for 15 min. Finally, the basilar membrane was washed thrice with 10 mM PBS. Morphologic changes in hair cells and stereocilia were observed in the basilar membrane-stretched preparation, and images were acquired using a Leica LSM 700 laser scanning microscope.

### 2.6. Scanning Electron Microcopy (SEM)

Myo3a mutant mice and wild-type mice were anesthetized using pentobarbital sodium and perfused with 4% PFA. The cochleae from Myo3a mutant and wild-type mice were removed, fixed with 2.5% glutaraldehyde in 0.1 M PBS at 4°C overnight, and decalcified in 10% EDTA [31]. The cochleae were dissected out from the temporal bone, and the stria vascularis, Reissner's membrane, and tectorial membrane were removed [32]. The organ of Corti was exposed and postfixed in 1% osmium tetroxide in 0.1 M phosphate buffer [33] for 2 h before dehydration in an ethanol series and critical drying point in an Autosamdri-815A (Tousimis) [32]. Samples were mounted with carbon tape, coated with gold, and imaged with a JEOL 7000 field emission gun scanning electron microscope.

### 2.7. FM1-43 Staining Experiments

FM1-43 staining experiments were performed as previously reported [34]. First, the Myo3a mutant and wild-type mice were anesthetized, and their cochleae were removed. Cochlear samples were bathed with 3 mM FM1-43 solution for 20 s. The cochlear samples were treated with 4% PFA at 4°C overnight. On the next day, the cochlear samples were washed with 10 mM PBS for 10 min for several times. The cochleae were then observed under an LSM 780 confocal microscope.

### 2.8. Noise Exposure

Nine 4.5-month-old Myo3a mutant mice and seven wild-type mice were anesthetized with 0.007 g/mL pentobarbital sodium by intraperitoneal injection (50 mg/kg body weight) and placed in a 24 cm × 24 cm × 18 cm stainless steel cage for full white noise handling. The loudspeaker was located just in front of the cage. Sound intensity was calibrated using a standard sound level meter prior to exposure to noise. The mice were continuously treated with 98 ± 2 dB SPL for 2 h to produce a temporary change in auditory threshold shifts.

### 2.9. Statistical Analysis

All data were expressed as mean ± SD, and all experiments were repeated at least thrice to ensure the data accuracy and repeatability. Statistical analyses were implemented using Microsoft Excel, and charts were constructed using GraphPad Prism 5 software. Two-tailed, unpaired Student's *t*-tests were used to determine statistical significance when comparing two groups. *P* < 0.05 was considered statistically significant [35].

## 3. Results

### 3.1. Generation of Myo3a Y137C Mice Using CRISPR/Cas9

**Figure 1:**
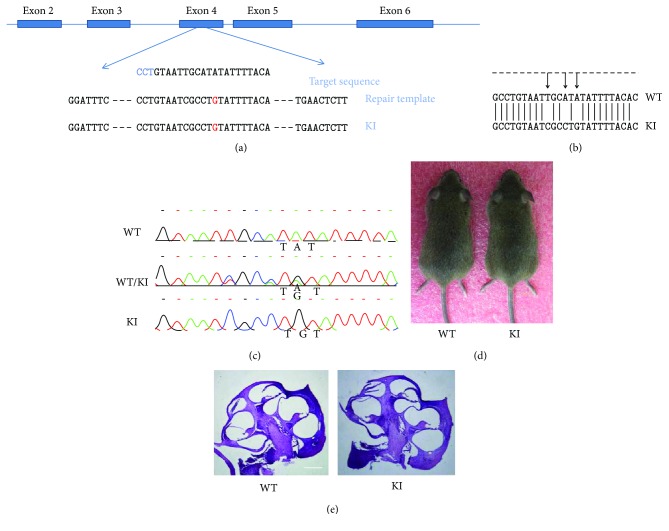
The generation of Myo3a mutant mice using CRISPR/Cas9. (a) Schematic diagram of targeting the mouse myosin IIIA gene. sgRNA was at exon 4 (indicated by the blue rectangles). The point mutation is in red. (b) The comparison of DNA sequences between Myo3a mutant mice (now referred to as Myo3a KI/KI mice) and wild-type mice. (c) Sequence of wild-type mice, heterozygous mice, and homozygous Myo3a KI/KI mutant mice. TAT was changed to TGT, demonstrating the missense mutation at mouse Y137C. (d) Gross morphology of Myo3a KI/KI and wild-type mice at the age of two months. There was no obvious difference. (e) Cochlea morphology is normal in Myo3a mutant mice. Hematoxylin and eosin (HE) staining showed no prominent difference between Myo3a mutant and wide-type mice cochlear at the age of two months. Scale bar = 20 *μ*m.

To mimic Myo3a Y129C mutation in human, we introduced the A410G (Y137C) mutation in mice by using CRISPR/Cas9 technology (Figure 1). To introduce the A410G mutation, we first induced a double-strand break (DSB) near the A410 and then repaired the DSB using the repair template with the point mutation of interest.

The sgRNA containing the 20 bp target sequence complexed with Cas9 protease can introduce DSB into the target sequence near a protospacer-adjacent motif (PAM) sequence. On the basis of this principle, we designed a specific sgRNA targeting the sequence near the A410 and cloned the sgRNA into the Px330 plasmid containing the Cas9 gene sequence. Thereafter, we designed a repair template with the mutation of interest based on the location of the DSB. The two synonymous mutations on the repair template aim to prevent a secondary targeting of sgRNA. The Px330 plasmid (5 ng/*μ*L) and the repair template (10 ng/*μ*L) were injected into the pronucleus of the fertilized mouse eggs. Exactly 113 fertilized eggs with clear pronucleus were injected, and 58 fertilized eggs with normal morphology after injection were transferred to the fallopian tubes of three pseudopregnant CD1 female mice. A total of 17 mice were born at 19 days after the transplantation. According to the PCR analysis results of the 17 mice, the mutation of interest was observed in 2 mice—a homozygous mouse and a heterozygous mouse (Figure 1(c)). We also performed sequencing at easy-off sites and found no off-targets. To obtain homozygous Myo3a KI/KI mice, we mated F0 mice with wild-type CBA/CaJ mice to generate F1 heterozygous mice, which were self-crossed to obtain homozygous Myo3a mutant mice. The Myo3a mutant mice were found to harbor cysteine in 137 rather than tyrosine in WT mice, similar to the mutation observed in humans.

Myo3a mutant mice were viable and fertile with no apparent abnormalities in their gross morphology (Figure 1(d)). We then examined the presence of an abnormal structure in the Myo3a mutant mice cochlea by paraffin sectioning and H&E staining. Structural abnormality was not observed in the Myo3a mutant mouse cochlea (Figure 1(e)).

### 3.2. Progressive Hearing Loss in Myo3a Mutant Mice

**Figure 2:**
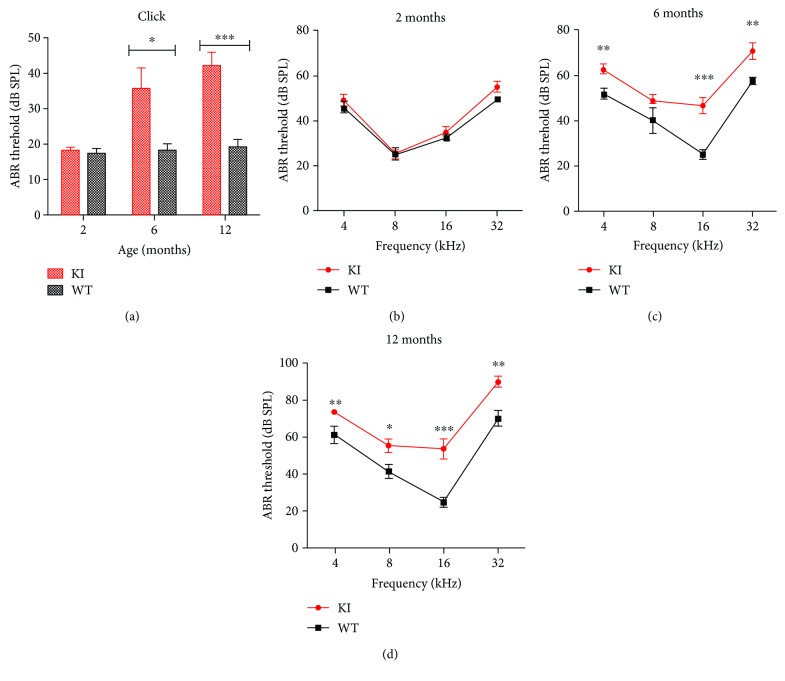
ABR analysis in Myo3a mutant mice (red) and wild-type mice (black) at two months, six months, and twelve months. (a) ABR measurements for broadband click. (b) Frequency-specific pure tone stimulation of Myo3a KI/KI mice and wild-type mice at two months old (b), six months old (c), and twelve months old (d). In contrast to wild-type mice, Myo3a KI/KI mutant mice showed progressive hearing loss. Compared with WT threshold at the corresponding frequency as determined by Student's *t*-test. ^∗^*P* < 0.05; ^∗∗^*P* < 0.01; ^∗∗∗^*P* < 0.001. Error bars indicate SEM. *n* > 5 for control and mutant mice for each experiment.

In humans, Myo3a mutation can cause nonsyndrome-type deafness. Thus, we wanted to test whether Myo3a mutant mice show the same symptoms. To determine whether Myo3a mutant mice demonstrate age-related deafness, we tested the hearing threshold of 2-, 6-, and 12-month-old Myo3a mutant and wild-type mice by ABR measurement. There results indicated no significant difference in the hearing threshold between 2-month-old wild-type (*n* = 6) and Myo3a mutant mice (*n* = 7) (Figure 2(b)). However, by the age of 6 months, Myo3a mutant mice (*n* = 7) exhibited a significantly higher hearing threshold than the wild-type mice (*n* = 6) (*P* < 0.05, Student's *t*-test) in both the click stimuli and at different frequencies (4, 16, and 32 kHz, Figures 2(a) and 2(c)). Hearing differences between the wild-type and Myo3a mutant mice were increasingly pronounced (*P* < 0.01) at 12 months in the broadband click and tone burst stimuli at frequencies of 4, 8, 16, and 32 kHz (Figures 2(a) and 2(d)). Therefore, we can conclude that Myo3a mutant mice exhibited a gradual hearing loss, which is similar to the characteristics of presbyacusis in humans.

### 3.3. Progressive Stereocilium Degeneration and Hair Cell Loss in Myo3a Mutant Mice

**Figure 3:**
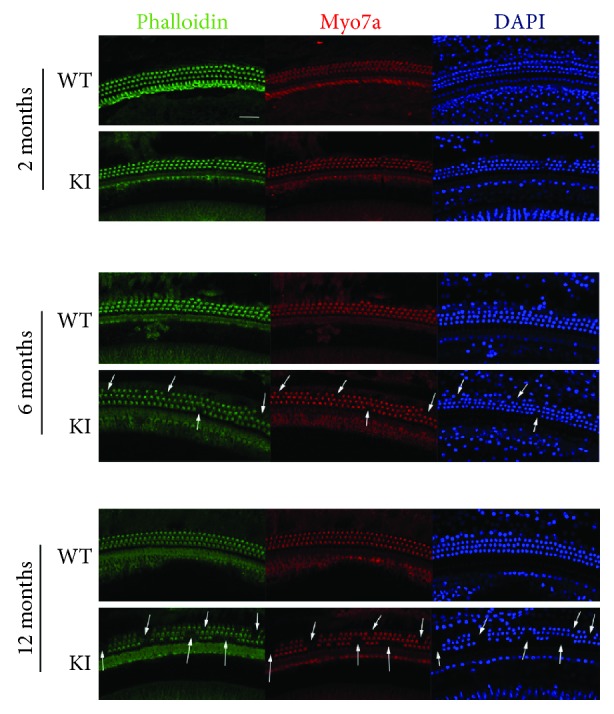
The degeneration of stereocilia and the hair cell loss in the Myo3a mutant mice showed by confocal images. Confocal images of the stereocilia, hair cells, and nucleus in Myo3a mutant and WT mice at two months old, six months old, and 12 months old. Images were taken from the middle turn of the cochlea. Scale bar = 20 *μ*m. The stereocilium degeneration and the loss of hair cells can be seen in the Myo3a mutant mice from 6 months old, and this phenomenon becomes more serious in 12-month-old Myo3a mutant mice.

We investigated the mechanism of senile deafness in Myo3a mutant mice and examined cochlear changes. We dissected the cochleae of 2-, 6-, and 12-month-old wild-type and mutant mice and used phalloidin, DAPI, and Myo7a to stain hair cell stereocilia, hair cell nucleus, and hair cells, respectively. Confocal images of the basilar membrane showed that the stereocilia and hair cells of the wild-type and Myo3a mutant mice were intact at the age of 2 months; by contrast, the stereocilia of the Myo3a mutant mice started to degenerate, and the hair cells began to disappear at 6 months. By the age of 12 months, stereocilium degeneration and hair cell loss became incrementally serious (Figure 3). The confocal image showed that stereocilium degeneration was consistent with hair cell loss.

**Figure 4:**
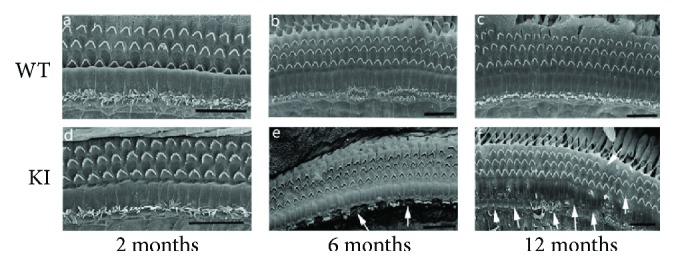
The degeneration of stereocilia in the Myo3a mutant mice showed by SEM. SEM images of the hair cells in Myo3a mutant and WT mice at two months old (a, d), six months old (b, e), and 12 months old (c, f). The inner hair cell stereocilium loss was found in 6-month-old Myo3a mutant mice (e), and this phenomenon becomes more serious in 12-month-old Myo3a mutant mice (f). Scale bar = 20 *μ*m.

**Figure 5:**
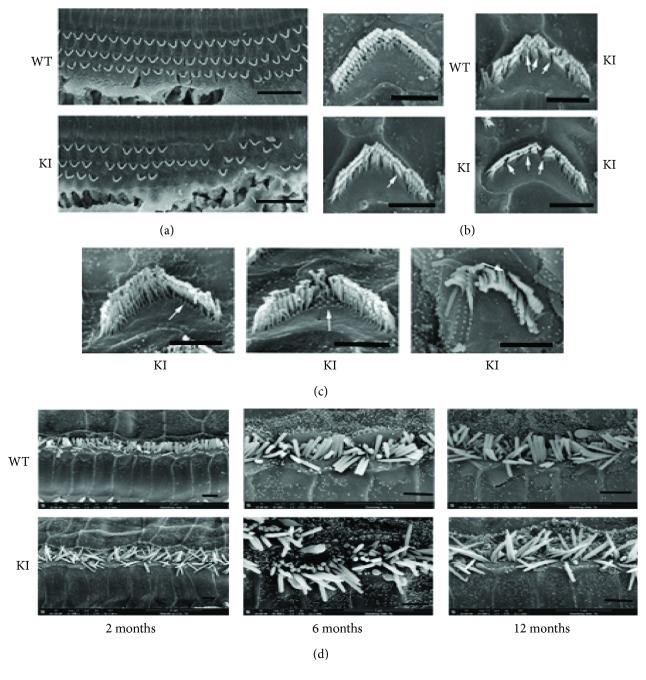
SEM images showed the abnormal structure of the stereocilia in Myo3a mutant mice. (a) The outer hair cell stereocilium loss was serious in 6-month-old Myo3a mutant mice. (b) Fusion phenomenon was observed in some stereocilia of mutant mice. (c) The stereocilia of some outer hair cells were found to be shorter, and the degeneration started from the innermost line of the stereocilia. (d) The stereocilia of inner hair cells become sharp in Myo3a mutant mice at the age of 2 months, 6 months, and 12 months. Scale bar = 10 *μ*m for (a) and 2 *μ*m for (b, c, and d).

To further verify the accuracy and reliability of the above result, we used SEM to observe the hair cells of mutant and wild-type mice in detail. Consistent with the above results, stereocilium degeneration and hair cell loss occurred in 6-month-old Myo3a mutant mice, especially in the stereocilia of inner hair cells (Figure 4(e)), whereas relatively complete hair cells and stereocilia were observed in wild-type mice (Figure 4(b)). By the age of 12 months, Myo3a mutant mice exhibited intensive stereocilium loss (Figure 4(f)), and this finding agreed with the ABR measurement. Through SEM, we also found structural abnormality in the remaining stereocilia in Myo3a mutant mice, in which the stereocilia of certain outer hair cells began to shorten and degenerate from the innermost line of stereocilia (Figure 5(c)). Fusion phenomenon was also noted in certain stereocilia (Figure 5(b)). The results were consistent with the expression location of Myo3a, in which morphological changes were also observed at the tips of the stereocilia in Myo3a mutant mice (Figure 5(d)). Most of the inner hair cells possess cuspidal stereocilia.

**Figure 6:**
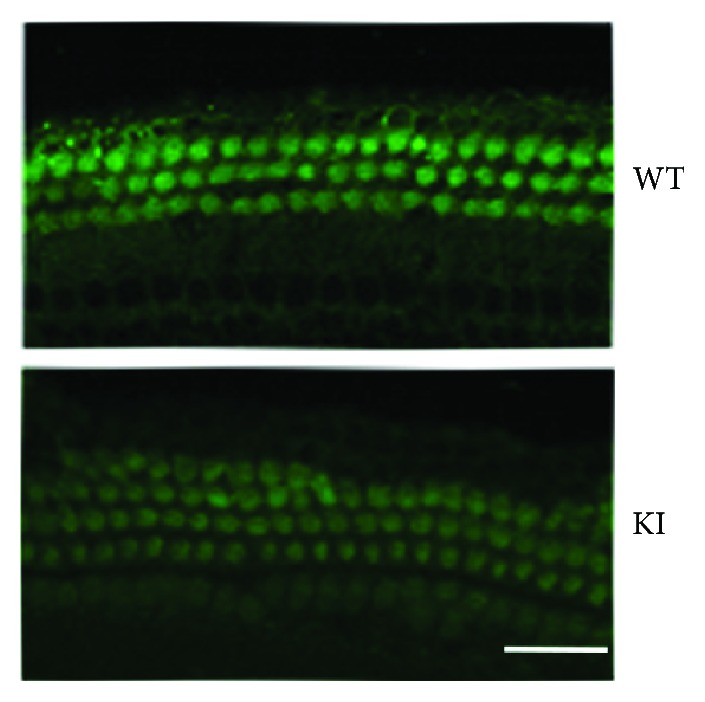
MET activity is not affected in Myo3a mutant mice. FM1-43 staining showed that the MET activity is normal in Myo3a mutant mice. Scale bar = 20 *μ*m.

During hearing formation, transformation from mechanical energy to electric energy is crucial, and ion channel plays a critical role in this process. The ion channel is located at the tip of the stereocilia where Myo3a is expressed. Thus, we investigated whether the function of the MET activity was affected in Myo3a mutant mice. We used the FM1-43 dye to stain the hair cells of wild-type and Myo3a mutant mice to determine whether the function of the MET activity was affected. The results are shown in Figure 6. Both the wild-type and Myo3a mutant mice showed positive FM1-43 staining in hair cells. This finding indicated that the MET activity of Myo3a mutant mice remained intact and that Myo3a is not a direct component of MET channels.

### 3.4. No Difference in Noise Resistance between Myo3a Mutant and Wild-Type Mice

**Figure 7:**
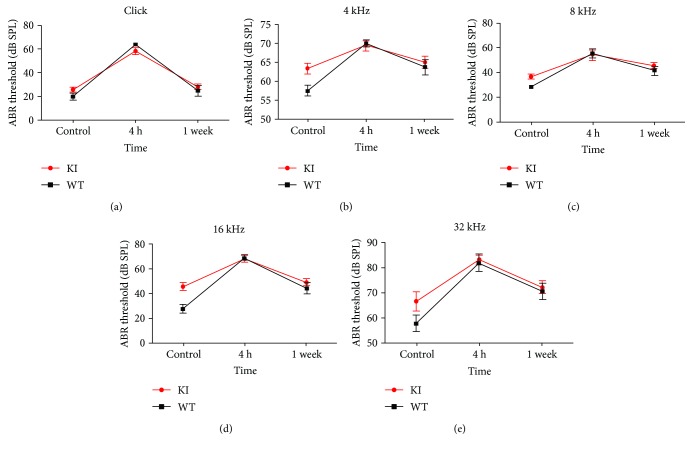
ABR analysis in Myo3a mutant and wide-type mice after noise exposure. ABR threshold was tested for broadband click (a) and frequency-specific pure tone (b, c, d, e) on Myo3a mutant and wide-type mice before (control), 4 h after, and 1 week after noise exposure (*n*≧7); error bars indicate SEM.

Presbyacusis is highly similar to noise-induced deafness in clinical pathology. Numerous senile deaf individuals are extremely sensitive to noise. To verify whether the noise resistance in the Myo3a mutant mice was affected, we performed continuous white-noise experiment for 2 h in 4.5-month-old wild-type and Myo3a mutant mice. Hearing test was conducted in wild-type and mutant mice before, at 4 h after, and at 1 week after noise treatment. The experimental results are shown in Figure 7; here, the line graph depicts no difference in noise resistance between Myo3a mutant and wild-type mice. The hearing threshold of the wild-type and Myo3a mutant mice significantly increased at 4 h after the white-noise experiment. Statistical analysis showed no difference in hearing between mutant and wild-type mice after the noise treatment. However, no difference in hearing was observed between the wild-type and Myo3a mutant mice. We repeated the noise experiment twice to verify the results. No difference in hearing was determined between the wild-type and Myo3a mutant mice after the noise treatment. Therefore, we conclude that the noise resistance of Myo3a mutant and wild-type mice was similar. However, there remains unanswered question. In our experiments, noise exposure seems to “erase” the initial difference in ABR thresholds between WT and KI, but the mechanism of this phenomenon is still unknown. We will perform further investigation in our future experiments.

## 4. Discussion

Using CRISPR/Cas9 technology, we generated a model of Myo3a Y137C mice consisting of a mutation in the kinase domain similar to that observed in the human Myo3a Y129C mutation. Through ABR hearing detection in mutant mice and wild-type mice, we found that the mutant mice exhibited progressive hearing loss. In 6 months, the hearing threshold of the mutant mice increased relative to that of the wild-type mice, whereas the hair cells of the 6-month-old mice started to degenerate. At 12 months of age, mutant mice exhibited significantly different hearing threshold and a more severe hair cell degeneration in the inner ear compared with that in the wild-type mice. The increased hearing threshold coincided with the loss of hair cells. This finding shows that kinase activity is crucial for the function of Myo3a and inner hair cells. Progressive hearing loss in Myo3a mutant mice was similar to that observed in human Myo3a mutants. Thus, the Myo3a mutant mouse model that we constructed was not only a good model for presbyacusis but also a satisfactory human disease model.

### 4.1. Kinase Domain Is Important to Myo3a

As a member of the myosin family, Myo3a transports cargo Espin1 to the top of the stereocilia [36]. In contrast to a previous mouse model that contains a stop codon at 1041 but maintains kinase activity, the mouse model in the current study involves mutation at the kinase domain of Myo3a. Our study has proved that kinase domain is important to the function of Myo3a.

Myo3a retains its motility by using the motor domain and the C-terminal THDII domain in combination with actin. In the wild-type mice, the THDI domain specifically binds to cargo Espin1, ensuring that Myo3a can transport Espin1 to the tip of the stereocilia to stabilize its structure [37]. The phosphorylation-dephosphorylation mechanism plays an essential role in normal Myo3a function [38]. Myo3a concentrates at the tip of the stereocilia, and the kinase domain interacts with and phosphorylates the motor domain because of high Myo3a concentration in the stereocilia; furthermore, motor activity is decreased by phosphorylation, and the phosphorylated motor domain loses its affinity with actin and tends to be transported back to the cell body to bind Espin1 again [39]. In the cell line, wild-type Myo3a localizes along the length of the filopodia, and the kinase-deleted construct present increases tip localization [13]; thus, Myo3a, which does not exhibit kinase activity, may demonstrate difficulty in returning to the cytoplasm to bind with Espin1 again.

Myo3a kinase activity may be disrupted in our mutant mouse model. Myo3a autophosphorylation cannot occur even at high Myo3a concentration at the top of the stereocilia so that the motor domain cannot be phosphorylated. The active motor domain tightly binds with actin and cannot be detached from actin. Thus, Myo3a that is located at the tip of the stereocilia cannot return to the cytoplasm and continue to transport Espin1. Thus, the structure of the top of the stereocilia is abnormal, thereby affecting the normal stereocilia functioning and causing progressive hearing loss.

### 4.2. Myo3b May Compensate for the Function of Myo3a

We observed normal hearing threshold and normal inner ear hair cell development in the 2-month-old Myo3a mutant mice. The elevated hearing threshold and hair cell degeneration of mutant mice presented at ≥6 months of age. The explanation why mutant mice exhibit progressive rather than profound hearing loss at a young age is unknown. We speculate that Myo3b compensates for the loss of Myo3a.

Compensatory effects in higher organisms are highly common. Myo3b localizes at cochlear hair cell stereocilium tips, similar with the localization of Espin1 and Myo3a [37]. Myo3b contains the motor domain and the THDI domain but lacks the THDII domain, which is contrary to Myo3a, which lacks the ability to “walk along” actin [37]. In COS7 cell line, Myo3a that lacks THDII-actin-binding domain does not localize to filopodial tips but moves toward the tip and promotes filopodial elongation when coexpressed with Espin1 Myo3a. This finding suggests that when lacking the THDII-actin-binding domain, Myo3a can bind to the cargo Espin1 with its THDI domain and then use its motor domain and the Espin1 ABM domain to travel along actin [36]. Similarly, when using the THDI domain combined with Espin1, Myo3b can obtain its capability to “walk along” actin by using its motor domain and the Espin1 ABM domain and perform the function of transporting Espin1 to the tip of stereocilia [37]. In an Espin1 knock-out mouse model, Myo3b is not detectable at the stereocilium tips of extrastriolar hair cells [40], demonstrating that Myo3b can transport Espin1 to the tip of stereocilia only when combined with Espin1. Nevertheless, Myo3b compensates for Myo3a for Espin1 shipping.

In Myo3a mutant mice, Myo3b can compensate for the loss of function of Myo3a because Myo3b is located at the stereocilium tip of the inner ear hair cells. Thus, we observed normal development of the inner ear hair cells. Myo3a and Myo3b double knock-out mice are profoundly deaf, demonstrating that class III myosins play redundant roles in hearing function [41].

Unlike a previous Myo3a knock-out mouse model [21, 41], the Myo3a mutant mice in the current study were maintained on the CBA/CaJ background. Our mouse model is suitable for studying DFNB30-type deafness in human because the B6 mice exhibit the characteristic of senile deafness.

## 5. Conclusion

The knock-in mice with Myo3a kinase domain mutation displayed progressive hearing loss and stereocilium degeneration in inner ear hair cells. Our mouse model of Myo3a point mutation made by CRISPR/Cas9 technology can simulate human diseases well and provide a good mouse model for the study of senile deafness.

## Data Availability

The data used to support the findings of this study are available from the corresponding author upon request.
